# Gate Control of the Current–Flux Relation of a Josephson Quantum Interferometer Based on Proximitized Metallic Nanojuntions

**DOI:** 10.1021/acsaelm.1c00508

**Published:** 2021-09-08

**Authors:** Giorgio De Simoni, Sebastiano Battisti, Nadia Ligato, Maria Teresa Mercaldo, Mario Cuoco, Francesco Giazotto

**Affiliations:** † NEST, Istituto Nanoscienze-CNR and Scuola Normale Superiore, I-56127 Pisa, Italy; ‡ Dipartimento di Fisica “E. R. Caianiello”, Universitá di Salerno, Fisciano, Salerno IT-84084, Italy; § SPIN-CNR, Fisciano, Salerno IT-84084, Italy; ∥ Department of Physics “E. Fermi”, Universitá di Pisa, Largo Pontecorvo 3, I-56127 Pisa, Italy

**Keywords:** Josephson effect, SQUID, superconducting magnetometer, gated metallic superconductor, proximity effect, SNS

## Abstract

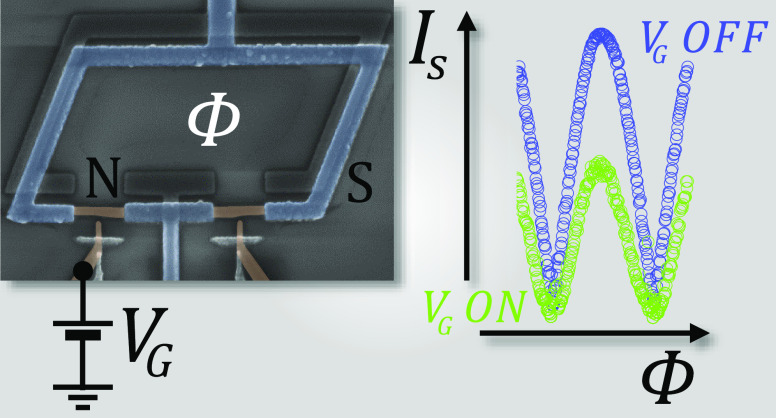

We demonstrate an Al superconducting quantum interference device in which the Josephson junctions are implemented through gate-controlled proximity Cu mesoscopic weak links. This specific kind of metallic weak links behaves analogously to genuine superconducting metals in terms of the response to electrostatic gating and provides a good performance in terms of current-modulation visibility. We show that through the application of a static gate voltage we can modify the interferometer current–flux relation in a fashion that seems compatible with the introduction of π-channels within the gated weak link. Our results suggest that the microscopic mechanism at the origin of the suppression of the switching current in the interferometer is apparently phase coherent, resulting in an overall damping of the superconducting phase rigidity. We finally tackle the performance of the interferometer in terms of responsivity to magnetic flux variations in the dissipative regime and discuss the practical relevance of gated proximity-based all-metallic SQUIDs for magnetometry at the nanoscale.

## Introduction

All-metallic gated superconducting transistors (GSTs) are a class of mesoscopic quantum devices, entirely realized with Bardeen–Cooper–Schrieffer (BCS) metals, in which the critical supercurrent (*I*_C_) can be largely regulated via electrostatic gating.^1−7^ Differently from proximitized semiconductors and low-charge-density superconductors^8−14^ where the critical current is controlled via conventional field-effect-driven charge-density modulation, in GSTs the *I*_C_ suppression is obtained, regardless of the sign of the gate voltage, without the carrier concentration being affected.^15^ The underlying physical mechanism has not been clearly identified yet, and a few hypotheses have been guessed to explain a plethora of experimental results, which cannot be comprehended in the bare framework of the BCS theory.^16^

Recently, a high-energy electron injection due to cold electron field emission from the gate has been claimed^17−20^ to have a major role in *I*_C_ suppression. This picture does not rely on novel physics. Yet, it does not seem compatible with some of the observed phenomenology such as the absence of a sum rule between currents originating from different gates,^4^ the response of in-vacuum suspended gated superconducting nanowires,^21^ and the nonthermal character of the switching current probability distributions of GSTs.^22,23^ A possible alternative^24−26^ explanation relies on the analogy between the creation of an electron–positron couple from the vacuum by a constant electric field in quantum electrodynamics (i.e., the so-called Sauter–Schwinger effect) and the creation of an excited condensate in a BCS superconductor. As another choice, the involvement of a voltage-driven orbital polarization at the surface of the superconductor has been proposed^27−29^ to be responsible for an unconventional phase reconstruction of the superconducting order parameter, leading to weakening and destruction of superconductivity. While high-energy electron injection due to field emission is likely to be strongly detrimental for preserving phase coherence in the superconductor, the two latter models are supposed to preserve it up to a large extent, and both predict the occurrence of a rotation of π in the macroscopic superconducting phase of the region affected by the gate voltage.

The information about the phase behavior of a superconductor subjected to the action of external stimuli can be experimentally accessed through a direct-current (DC) superconducting quantum interference device (SQUID):^30^ a superconducting ring interrupted by two Josephson weak links in parallel. A magnetic field threading the loop controls the current vs voltage (*IV*) characteristics of the SQUID via magnetic flux quantization^31,32^ and the DC Josephson effect,^33^ thus resulting in a modulation of the amplitude of the critical supercurrent. The impact of the electrostatic gating on the superconducting phase of a BCS superconductor was investigated so far only in monolithic Ti interferometers based on gated Dayem bridges.^34^ Such systems allowed to retrieve a footprint of the action of the gating on the switching current (*I*_S_) vs flux (ϕ) relation of the SQUID. Nonetheless, because of the large value of the SQUID inductance, the *I*_S_(ϕ) of these interferometers exhibited poor modulation visibility, with a significant deviation from the ideal sinusoidal behavior.^30^ This limited the access to a detailed information about the dependence of the current vs phase relation of gated metallic Josephson weak links on the applied voltage.

Here we investigate the impact of electrostatic gating on the current–phase relation of metallic mesoscopic Josephson junctions by demonstrating a SQUID in which the Josephson junctions are implemented through gate-controlled Cu weak links. These can carry a dissipationless phase-dependent supercurrent thanks to the proximity effect^35^ induced by the superconducting Al forming the interferometer ring.^36−38^ Gated superconducting/normal-metal/superconducting (SNS) proximitized Josephson weak links, based on Al/Cu/Al junctions, were recently demonstrated to behave analogously to genuine superconducting metals in terms of the response to electrostatic gating.^39^ Furthermore, these kinds of weak links possess typically a Josephson inductance significantly larger than superconducting Dayem bridges, securing a good performance in terms of current-modulation visibility.^36−38^ For the above reasons, we selected such a system as the suitable candidate to explore the impact of gating on the current–phase relation of a metallic weak link. Specifically, we show that the application of a constant gate voltage results in a strongly modified SQUID current–flux relation that might be compatible with the occurrence of a frustration of the superconducting phase due to activation of π-domains within the weak link. In addition, we discuss the performance of gated proximity-based all-metallic SQUIDs in terms of responsivity to magnetic flux variations in the dissipative regime.

## Effect of Gate Voltage on the SQUID Current–Flux Relation

**Figure 1:**
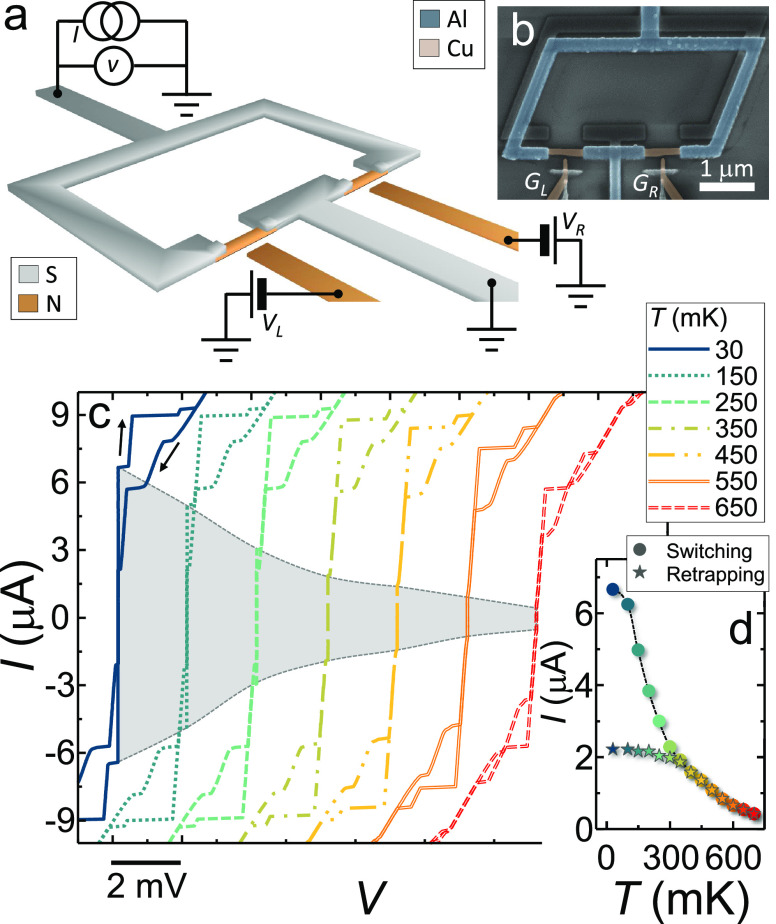
Proximity-based gated all-metallic DC SQUID. (a) Scheme of a DC superconducting quantum interference device (SQUID) based on superconductor/normal metal/superconductor (SNS) gated proximity Josephson junctions. The four-wire electrical setup is also shown. The left (L) and right (R) gates are polarized with voltage *V*_L_ and *V*_R_, respectively. (b) False-color scanning electron micrograph of a representative gated SNS SQUID. The Al interferometer ring is colored in blue. The Cu Josephson weak links and the left (*G*_L_) and right (*G*_R_) are colored in orange. (c) Current (*I*) vs voltage (*V*) forward and backward characteristics of a representative SNS SQUID at selected temperatures between 30 and 650 mK and at magnetic flux ϕ = 0.135ϕ_0_. Curves are horizontally offset for clarity. The *I*–*V* region corresponding to the presence of a Josephson current is colored in gray. Error on the voltage drop measure is lower than the width of the curves. (d) Switching (dots) and retrapping (stars) current of the same device of panel c vs temperature *T*. The difference between switching and retrapping current stems from heat generated in the junctions when approaching the superconducting state from the dissipative regime. The uncertainty on the measure of the switching and retrapping current is lower than the dot size.

Our gate-controllable superconducting interferometers (SNS SQUID) consist of a 100 nm thick Al superconducting loop interrupted by two Al/Cu/Al planar gated junctions. The loop of the SQUID spans a surface of about 7.5 μm^2^. Aluminum shows a strong proximization capability over copper, thanks to the good quality of the interfaces formed between these two metals.^39^ Furthermore, we emphasize that although higher critical temperature metallic superconductors, such as for example Nb or V, would certainly provide a stronger proximization of the normal metal domains,^36,37^ because of their high melting temperature, they turn out to be impractical in terms of ease of fabrication and of compatibility with our metal deposition technique. The Cu normal-metal wire was 120 nm wide, 630 nm long, and 20 nm thick. The weak links operate in the diffusive regime and within the long-junction limit, holding when the Thouless energy of the junction  μeV ≪ Δ_Al_ ≃ 180 μeV, where *D* ≃ 0.008 m^2^/s is the Cu diffusion coefficient,^39^*L* the weak-link length, and Δ_Al_ the superconducting gap of the Al banks. Moreover, two 80 nm wide Cu gate electrodes, labeled G_L_ and G_R_, were separated from the normal-metal wire by a distance of about 60 and 45 nm, respectively (in the representative device whose data are discussed in the following). Further details of the fabrication process are reported in the Methods section. A 3-dimensional representation of a typical SNS SQUID comprising the scheme of the four-wire electrical setup is displayed in Figure 1a, whereas a false color scanning electron micrograph of a representative device is shown in Figure 1b.

Figure 1c shows the *IV* characteristics of a representative SNS SQUID collected at several temperatures ranging from 30 to 650 mK. The curves are horizontally offset for clarity. For temperatures smaller than 750 mK, the *IV*s exhibit a clear Josephson effect with a switching current *I*_S_ of ∼7 μA at 30 mK and a normal-state resistance *R*_N_ ∼ 50 Ω. Because of electron heating in the normal state,^40,41^ the usual hysteretic behavior is observed when the *IV* is measured forward and backward with a retrapping current *I*_R_ ∼ 2 μA at 30 mK. A plot of the switching and the retrapping current vs temperature (*T*) is shown in Figure 1d. As routinely observed in similar systems,^40,41^ the difference between *I*_S_ and *I*_R_ decreases by increasing *T* and vanishes at *T* ∼ 350 mK, thanks to the enhancement of thermal conductance of the junction superconducting electrodes and of the electron–phonon coupling,^42^ which allow for an efficient dissipation of the Joule power produced in the weak links.

**Figure 2:**
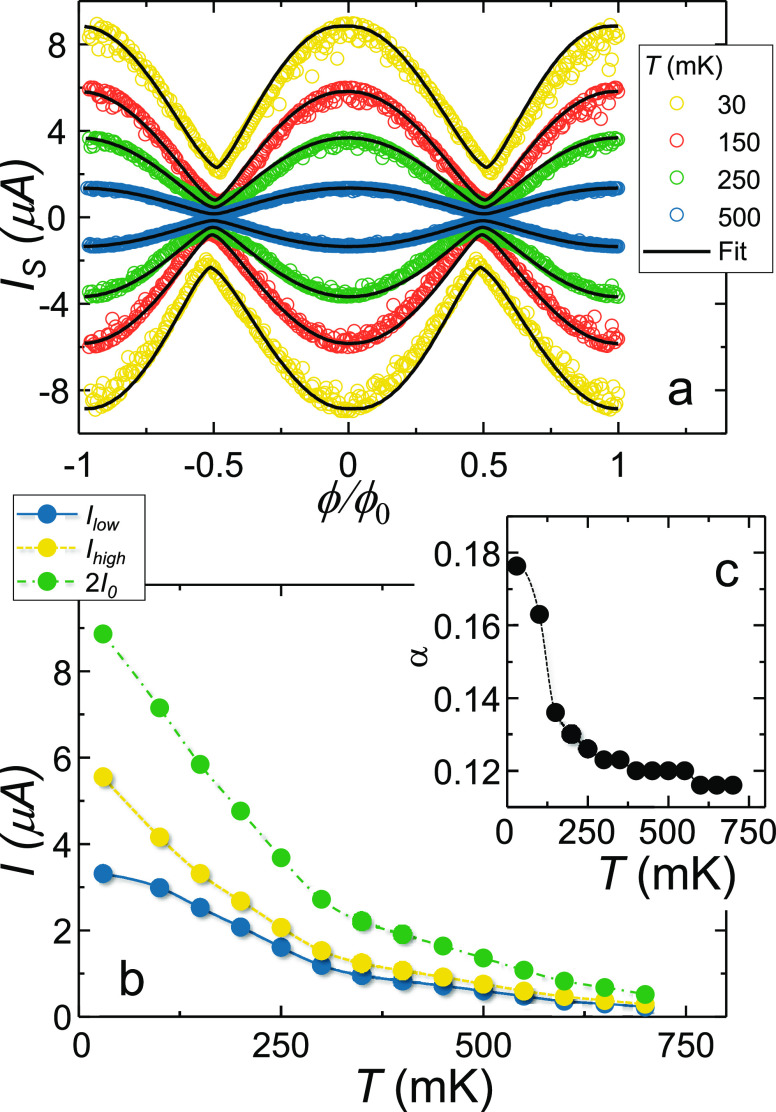
Switching current vs flux characterization of the SNS SQUID. (a) Switching current *I*_S_ of the SNS SQUID as a function of the external magnetic flux ϕ. ϕ was applied through a superconducting electromagnet. *I*_S_(ϕ) is shown for selected temperatures ranging between 30 and 500 mK. The uncertainty on the measure of the switching current is lower than the dot size. Superimposed on experimental data we show (black solid lines) the result of the fit obtained with the RSJ model. (b) Plot of the maximum switching current of the SNS SQUID (2*I*_0_) vs temperature (green dots). In the same plot we also show the critical current of the two junctions extracted through the RSJ fit. The value of the lowest of the two critical currents (*I*_low_) is represented by blue dots, whereas the value of the highest of the two critical currents (*I*_high_) is represented by yellow dots. (c) Asymmetry parameter (α) as a function of temperature. This value is extracted through the fitting procedure (see text).

To study the *I*_S_(ϕ) characteristics of the SNS interferometers, we measured their *IV*s as a function of the external magnetic field threading the SQUID loop. The device switching current was then extracted from the *IV*s to build the *I*_S_ vs ϕ curves. The *I*_S_(ϕ) of the device is reported in Figure 2a for selected temperatures between 30 and 500 mK, where ϕ_0_ ≃ 2.067 × 10^–15^ Wb is the magnetic flux quantum. For each temperature we plot both the positive (*I*_S_+__) and negative (*I*_S_–__) switching current branches, defined accordingly to the scheme of Figure 1a. By defining the modulation amplitude Δ*I*_C_ = *I*_MAX_ – *I*_MIN_ and the modulation average value ⟨*I*⟩ = (*I*_MAX_ + *I*_MIN_)/2 (where *I*_MAX_ and *I*_MIN_ are the maximum and minimum value of *I*_S_+__, respectively), a modulation visibility Δ*I*_S_/⟨*I*⟩ ∼ 90% is observed at 30 mK. Such a value is on par with the performance of state of the art SNS interferometers.^36,38^ Furthermore, it is about 9-fold higher than in gated monolithic Ti SQUIDs.^34^ When the temperature is increased, both ⟨*I*⟩ and Δ*I*_S_ decrease due to weakening of the proximity effect in the weak links, as routinely observed in these systems.

The modulation visibility is mainly determined by the difference between the critical currents of the two junctions. The latter can be extracted by fitting the *I*_S_(ϕ) data against the static zero-temperature resistively shunted junction (RSJ) model^30^
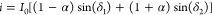
1
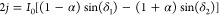
2
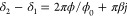
3where δ_1,2_ are the phase differences across the weak links; *i* and *j* are the supercurrent passing through and circulating in the SQUID, respectively. Within this formalism, defining , the asymmetry between the critical currents of the two junctions is accounted for. At fixed magnetic flux, *I*_S_+__ and *I*_S_–__ are defined as proportional respectively to the maximum and minimum values of *i* over all the values of δ_1_ and δ_2_ satisfying eqs 1–3, via the coefficient , corresponding to one-half of the maximum supercurrent of the SQUID as a function of ϕ. This model accounts also for the inductance  of the SQUID, through the screening coefficient . Although the RSJ model was conceived for tunnel-like Josephson junctions, it retains its validity also for SNS weak links that, like ours, fall in the *long* junction limit. A detailed description of the fit procedure is reported in the Methods section. The fit curves are shown on top of experimental data in Figure 2a (solid black lines). The good agreement between the RSJ model and experimental data is quantitatively confirmed by the coefficient of determination *R*^2^ of the fits, which ranges from 0.996 (at 500 mK) to 0.97 (at 30 mK). The value for 2*I*_0_ determined through the fitting procedure is plotted against the temperature in Figure 2b. Furthermore, we extracted the α parameter, which is reported in Figure 2c. α reaches the maximum value of ∼0.2 at 30 mK and decays when the temperature is increased. From α it is also possible to deduce the value of the critical currents of the two weak links, which are *I*_high_ ∼ 6 μA and *I*_low_ ∼ 3 μA for the junction with the higher and the lower critical supercurrent, respectively. A plot of *I*_high_ and *I*_low_ as a function of the temperature is reported in Figure 2b. The value for β derived from the fit is around 0.01 for every temperature, thereby confirming the negligible inductance contribution provided by the Al loop.

**Figure 3:**
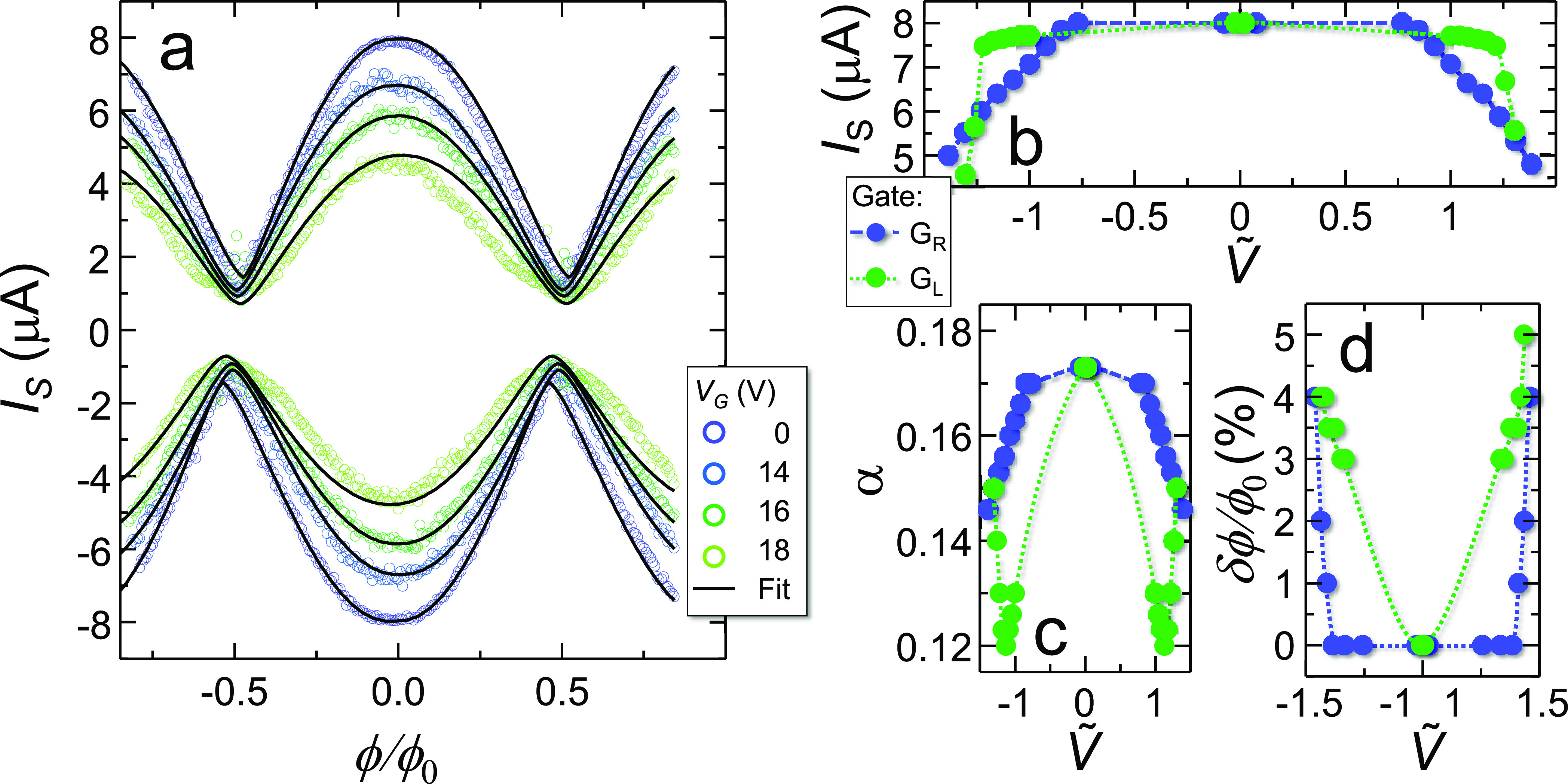
Electrostatic control of the current–flux relation in a gated SNS SQUID. (a) Positive (*I*_S_+__) and negative (*I*_S_–__) switching currents vs external flux of the SNS SQUID when a gate voltage is applied to one of the two junctions (*G*_R_). Curves are shown for gate voltage *V*_R_ between 0 (unperturbed case) and 18 V. The minima of the interference pattern are almost locked within the explored voltage range. The maxima are dumped by a factor reaching ∼0.5 at 18 V (green dots). Superimposed to experimental data, we show (black lines) the result of the RSJ fit. At 18 V a significant deviation from the conventional single-tone behavior in the current–flux relation is observed. The same qualitative behavior was observed when the left gate was polarized and for negative values of the gate voltage. The error on the measure of the switching current is lower than dot size. (b) Maximum of the switching current of the SQUID vs normalized gate voltage *Ṽ* applied either to the left (green dots) or right (blue dots) gate electrode. The normalization factors are 14 and 46 V for the right and left gate, respectively, and correspond to a 10% suppression of the maximum switching current. (c) Asymmetry parameter α as a function of the normalized gate voltage *Ṽ* applied to the left (green) or right (blue) gate. (d) Plot of the additional phase shift (δϕ) introduced in the flux quantization relation (see eq 3) vs normalized voltage applied to the left (green) or right (blue) gate electrode.

To investigate the impact of the gate bias on the SNS SQUID current–flux relation, we measured *I*_S_(ϕ) when several values of gate voltage were independently applied to either the left and right gate electrode. Figure 3a shows the modulation patterns of *I*_S+_ and *I*_S–_ for different positive values of gate voltage *V*_R_ applied to *G*_R_ measured at 30 mK. *G*_L_ was left grounded. It is worth discussing several interesting gate-dependent features emerging from the data. *I*_MAX_ is constant up to about 12 V. Above this threshold it is suppressed by further increasing *V*_R_ and exhibits the same reduction for positive and negative gate voltages as well as for positive and negative current bias. The same qualitative behavior was observed by polarizing the left gate electrode, which due to a larger gate–junction distance was effective at higher voltages. Furthermore, the left weak link exhibited a steeper variation of the critical current in response to the gate voltage. This behavior might be ascribed to a difference between the Thouless energy of the weak links, stemming from slight geometrical differences. Indeed, *E*_Th_ was observed to be one of the parameters determining the responsivity of SNS weak links to the gate voltage.^39^ The SQUID switching current as a function of gate voltage applied alternatively to *G*_L_ or *G*_R_ is shown in Figure 3b against *Ṽ*, i.e., the voltage normalized to the values at which the switching current was suppressed by 10%. This equals 14 and 46 V for *G*_R_ and *G*_L_, respectively.

In stark contrast to the conventional *T*-dependent case, in which both minima and maxima of the modulation pattern converge to 0 by enhancing the temperature, in the gate-dependent case the amplitude of the minima of *I*_S+_ (and the maxima of *I*_S–_) are apparently almost locked in the explored voltage range. We start our discussion on such an unconventional phenomenology by recalling that following from eqs 1 and 3
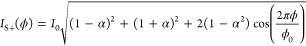
4This expression, which holds when β is negligible, allows to derive the *I*_S_(ϕ) extremal values *I*_MAX_ = 2*I*_0_ = *I*_L_ + *I*_R_ and *I*_MIN_ = 2α*I*_0_ = |*I*_L_ – *I*_R_|. These relations imply that it is not possible to affect *I*_MAX_ (which in our data is suppressed by an ∼50% factor) keeping *I*_MIN_ constant unless *I*_L_(*V*_R_ = 0) – *I*_L_(*V*_R_) = *I*_R_(*V*_R_ = 0) – *I*_R_(*V*_R_) [and *I*_R_(*V*_L_ = 0) – *I*_R_(*V*_L_) = *I*_L_(*V*_L_ = 0) – *I*_L_(*V*_L_)] for each value of *V*_R_ (and *V*_L_). This condition is not only extremely unlikely to be satisfied, but it seems also incompatible with the typical length scale of the gating effect in metallic superconductors. Indeed, it was shown^1,17^ that the critical current suppression due to the application of a gate voltage exponentially decays with the distance from the gate itself. In other words, gating is a *local* effect, which, acting on just one of the weak links, can affect *nonlocally* the response of the whole SNS SQUID.

To further elaborate on the above question, we believe interesting to discuss the results of the RSJ fitting of the *I*(ϕ)s obtained at different gate voltage values (see black lines in Figure 3a). The fit was performed by exploiting the same technique of the temperature-dependent case, but now including an additional phase shift (δϕ) in eq 3 such that δ_2_ – δ_1_ = 2πϕ/ϕ_0_ + πβ*j* + δϕ. The introduction of the latter parameter was necessary to successfully fit the *I*_S±_(ϕ) obtained for *V*_R_ > 15 V (and for *V*_L_ > 50 V). δϕ is plotted as a function of the gate voltage applied to either *G*_L_ or *G*_R_ in Figure 3d, while α(*Ṽ*) is plotted in Figure 3c. For |*Ṽ*| ≲ 1, the agreement between fit and data is optimal, with *R*^2^ ranging between 0.98 and 0.99. Above this threshold, however, the ability of the RSJ model to represent the current–flux characteristics progressively weakens: in particular, at |*Ṽ*| = 1.3 (equivalent to *V*_R_ = ±18 V for the data represented in Figure 3a), the deviation from the sinusoidal behavior is particularly evident. This behavior may be ascribed to a gate-induced modification of the Josephson current–phase relation of the weak link, which is driven out from the conventional monochromatic regime and turns out to be *colored* with additional higher-harmonic terms. In the same voltage range, δϕ increases, reaching the maximum value of ∼0.04 ϕ_0_ at |*Ṽ*| ∼ 1.5. It is also worthwhile to discuss the evolution of α(*Ṽ*), which monotonically decreases for increasing values of |*V*_R_| (blue dots in Figure 3c), while it shows minima for *Ṽ* ∼ ±1 when *G*_L_ is polarized (green dots in Figure 3c). In the framework of the RSJ model, such a behavior is accounted for through a simultaneous modification of the critical currents of both the weak links, and through the introduction of an additional phase shift term. We stress that this characteristics is not compatible with a *local* action of the gate voltage on the amplitude of the current–phase relation of the gated weak link. Indeed, if this were the case, on the one hand, by gating the weak link with the highest critical current, α should vanish (when *I*_high_ = *I*_low_) and then increase up to 1. On the other hand, α is expected to monotonically converge to 1 when *I*_low_ is suppressed due to the enhancement of asymmetry between the two junctions. For this reason we hypothesize a voltage-driven modification of the phase drop in the gated weak link. This then affects also the other weak link, and therefore the whole SQUID current–flux relation, through the flux quantization relation.

## Phase Frustration through π-Domain Activation

**Figure 4:**
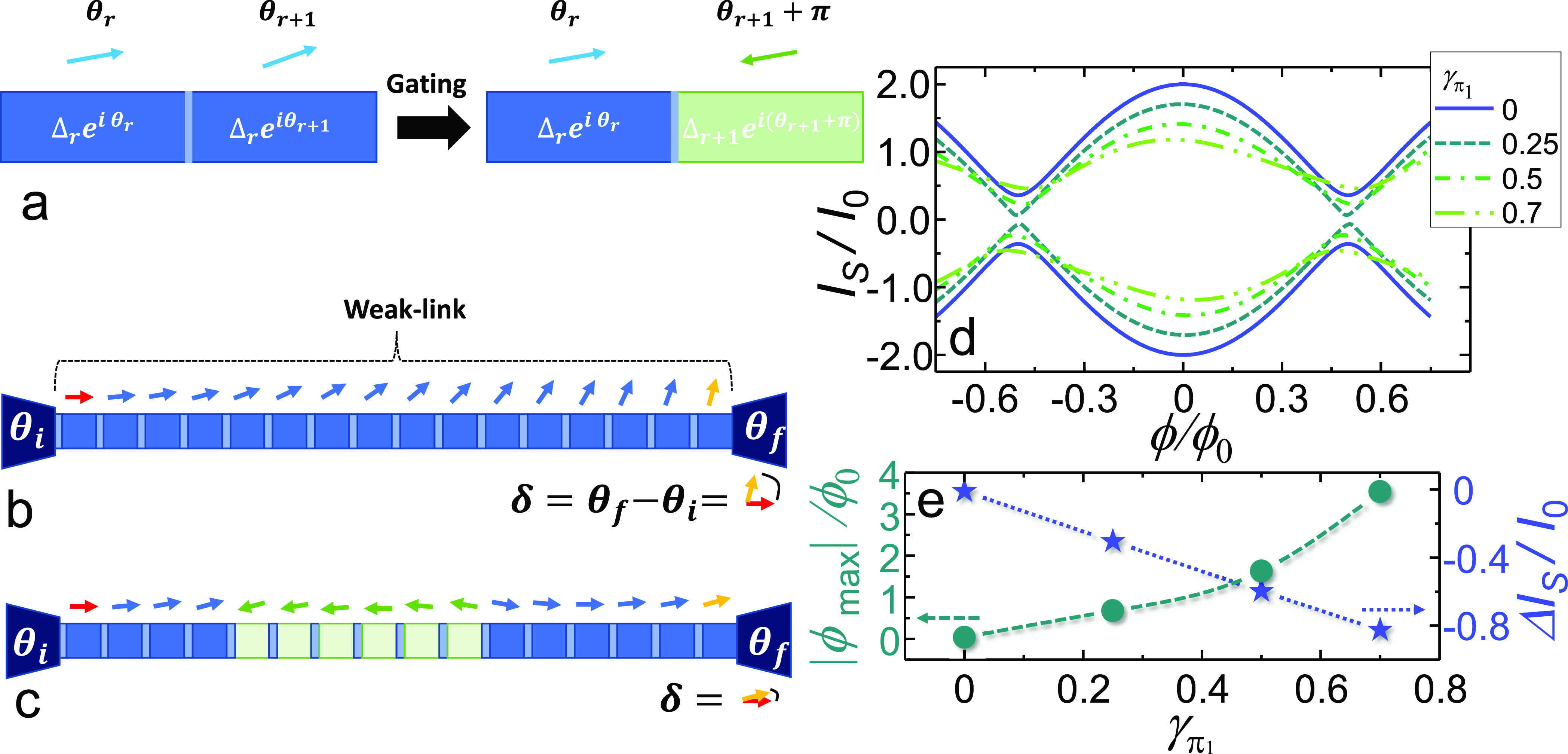
Gate-driven phase frustration through π-rotation. (a) Pictorial representation of the π-rotation mechanism induced by the gate voltage. The superconductor is represented through a one-dimensional chain of domains (blue blocks); each of them can be described by a generic complex order parameter Δ_*r*_e^*i*θ_*r*_^, where *r* is a domain index and θ_*r*_ is the superconducting phase in each domain. Under the action of the gate voltage we assume the phase of some of the domains to be rotated by π (green block). (b) When a supercurrent is injected through the weak link, the phase drop (δ) built across the latter results from the accumulation of phasor rotations acquired at each domain. (c) When a gate voltage is applied, a fraction of the domains proportional to the intensity of the electric field acquire a phase rotation of π with respect to 0 V case. The resulting phase drop over the weak link turns out to be frustrated due to the counter-rotation acquired by the phasor in the π-domains. (d) *I*_S_(ϕ) calculated through eq 6 with *N* = 2, *I*_1_ = 1.18, and *I*_2_ = 0.82. The latter values correspond to an asymmetry parameter α = 0.18, i.e., equivalent to that of our SNS SQUID. The amplitudes of 0-phase harmonics γ_0_1__ and γ_0_2__ were respectively set to 1 and 0 to recover the conventional sinusoidal monochromatic behavior when no gate voltage is applied. We show curves obtained for γ_π2_ = 0.05γ_π_1__ and for selected values of γ_π_1__ ranging between 0 and 0.7. The former case corresponds to an unpolarized gate voltage. (e) Phase shift of the maxima of *I*(ϕ), ϕ_MAX_, as a function of γ_π_1__ (left axis). The reported values are consistent with the result of the RSJ model for the experimental data for the parameter δϕ. We also show the γ_π_1__ dependence of the normalized variation of the maximum value of the interference pattern Δ*I*_S_/*I*_0_ (right axis).

We now discuss a possible phenomenological model based on the assumption that the gate voltage can affect only the phase of each superconducting domain composing the weak link and rotating it by a factor of π (see Figure 4a). This hypothesis assumes the existence of a fully coherent mechanism that can account for all the main features observed in gate-controlled *I*_S_(ϕ)s. Because of the polycrystalline nature of the copper wire forming our weak-links, we describe each domain through an order parameter Δ_*r*_ e^*i*θ_*r*_^, where Δ_*r*_ and θ_*r*_ are the amplitude of the gap and phase of the *r*th domain, respectively.^16^ In this framework, when a supercurrent is injected through the weak link, the phase drop δ built across the latter results from the accumulation of the phasor rotations acquired at each domain (see Figure 4b). In this condition, the current–phase relation of the weak link can be described by the conventional Josephson equation *I* = *I*_1_ sin δ, where *I* is the biasing current. When a gate voltage is applied, a fraction of the domains proportional to its intensity acquire a phase rotation of π (see Figure 4c) with respect to the unperturbed value. The phase drop over the weak link in this configuration is therefore overall *frustrated* due to the counter-rotation acquired by the phasor in the π-domain (green blocks in Figure 4b). This physical intuition finds a mathematical representation by modifying the weak-link current phase relation as follows:
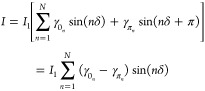
5where we recover the most general functional form^43^ by including an arbitrary number *N* of 0-phased and π-phased harmonics with weight γ_0_*n*__ and γ_π_*n*__, respectively, determined by the contribution of the π domains to the resulting phase drop. Following from this assumption, the RSJ current–flux relation of the SQUID modifies into
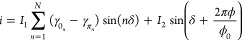
6where *I*_1_ and *I*_2_ account for the amplitude of the critical current of gated and nongated weak link, respectively. Figure 4d shows the *I*_S_(ϕ/ϕ_0_) calculated through this model with just two harmonics (*N* = 2) and with *I*_1_ = 1.18 and *I*_2_ = 0.82. The latter values correspond to an asymmetry parameter α = 0.18, i.e., compatible to that of our SNS SQUIDs. The amplitudes of 0-phase harmonics γ_0_1__ and γ_0_2__ were set respectively to 1 and 0 to recover the conventional sinusoidal *monochromatic* behavior when no gate voltage is applied. We show curves obtained for γ_π_2__ = 0.05γ_π_1__ and γ_π_1__ ranging between 0 (blue curve in Figure 4d) and 0.7 (light-green curve). The former case corresponds to a vanishing gate voltage. By increasing γ_π_1__, we mimic the action of the gate voltage, which amplifies the weight of the π terms for both the harmonics, thereby resulting in a suppression of the maxima of the current–flux relation (star plot in Figure 4d). The latter reaches a value of ∼50% for γ_π_1__ = 0.7. Besides, *I*(ϕ) minima undergo a nonmonotonic and much more limited variation. We wish to emphasize that by introducing just one additional harmonic, we obtained a significant deviation from the sinusoidal behavior, which resembles that of the experimental data. Furthermore, the shift of the maxima of *I*_S_(ϕ) (dots in Figure 4d) are consistent with the result of the RSJ fit procedure for the parameter δϕ, reaching a value of ∼4% (see Figure 3d for a comparison).

## Effect of Gating in the Dissipative Regime

Among available magnetic field sensors, SQUIDs are the devices of choice for those applications requiring ultrahigh sensitivity at the nanoscale. SQUIDs have progressively become an essential tool for probing several systems, such as magnetic molecules and nanoparticles, single electrons, and cold atom clouds. Beyond the detection of magnetic moments (down to the single spin resolution), SQUIDs play in a front row role in a vast field of applications ranging from microbolometry^44^ and spintronics to drug delivery and cancer treatment.^45^ In this last section we discuss the performance of our SNS SQUID in view of its possible exploitation as a gate-tuned magnetic flux sensors operating in the dissipative regime. The latter is conventionally obtained by current biasing the interferometer above its critical current. Variations of the magnetic field threading the loop translate into variations of the voltage drop (*V*) developed across the Josephson junctions.

**Figure 5:**
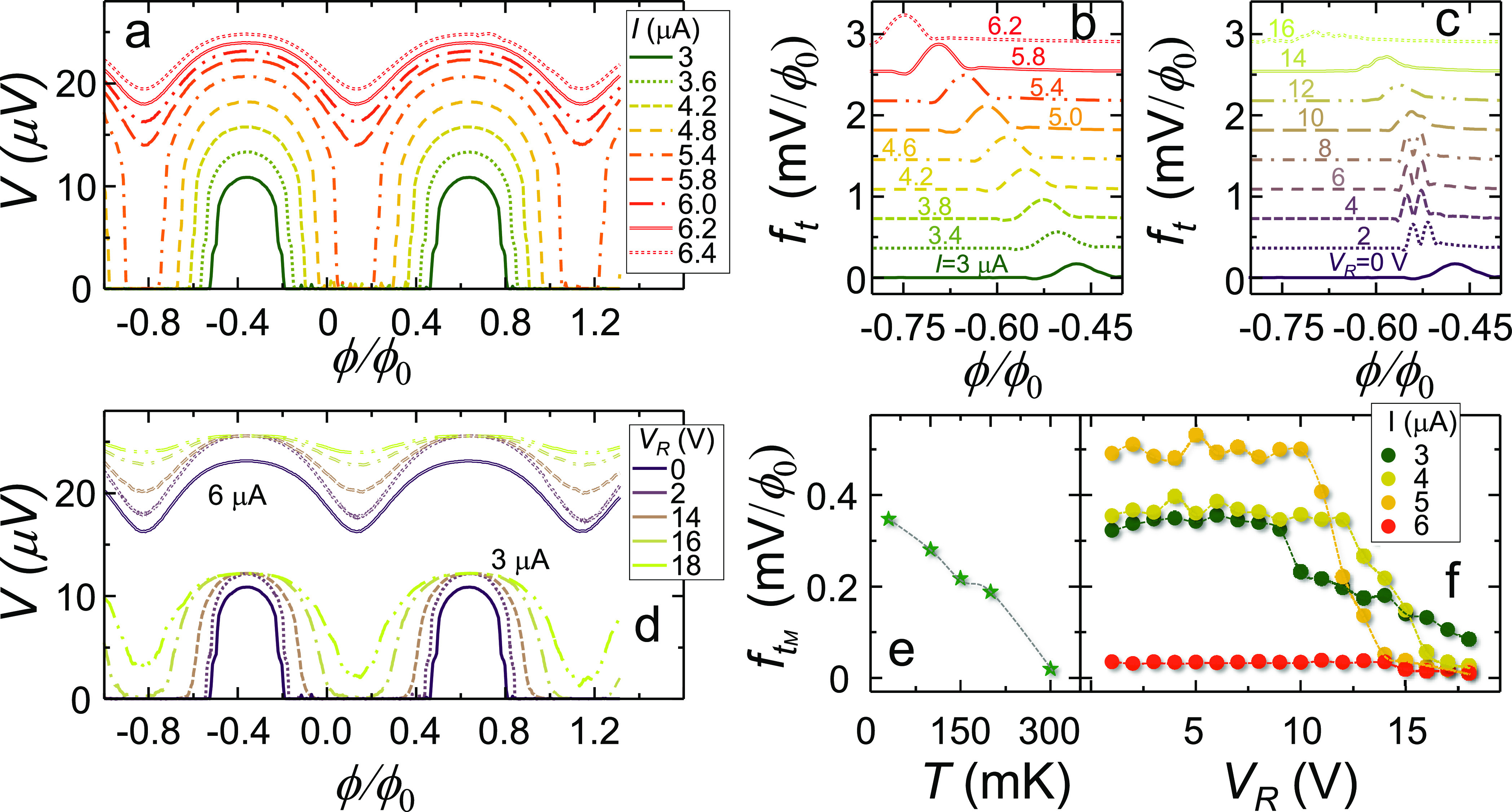
Effect of gating in the dissipative regime. (a) *V*(ϕ) characteristics at 30 mK for selected bias current values between 3 and 6.4 μA. The measurements were performed with a standard four-wire lock-in technique by biasing the device via a 17 Hz sinusoidal current signal. Below *I* ≃ 6 μA, the curves exhibit a zero voltage drop for magnetic fluxes such that *I* < *I*_C_(ϕ). A finite *V* value is instead measured when the device switches into the dissipative regime due to bias current being higher than the flux-dependent critical current. (b) Transfer function *f*_*t*_ vs ϕ for selected amplitudes of the biasing current. *f*_*t*_ was calculated through numerical differentiation of the *V*(ϕ) characteristics measured at 30 mK. (c) Transfer function *f*_*t*_ vs ϕ for selected values of *V*_R_ at 30 mK. (d) *V*(ϕ) curves obtained for *I* = 3 μA (single lines) and *I* = 6 μA (double lines) and for *V*_R_ ranging between 0 and 18 V. (e) Maximum value of *f*_*t*_ (*f*_*t*_*M*__) vs T. (f) *f*_*t*_*M*__ vs *V*_R_ for selected values of the biasing current at 30 mK. The error on plots in panels a–d is lower than line widths. The uncertainty on the measure of *f*_*tM*_ in panels e and f is lower than the dot size.

Figure 5a shows the *V*(ϕ) curves measured at 30 mK on a representative device by the four-wire lock-in technique for selected amplitudes of the 17 Hz sinusoidal current-bias signal *I*. Below *I* ∼ 6 μA, the curves exhibit a zero voltage drop for magnetic fluxes such that *I* < *I*_S_(ϕ). A finite *V* value is instead measured when the interferometer switches into the dissipative regime for the biasing current being higher than the flux-dependent switching current. This results in a strongly nonlinear behavior at the switching points, corresponding to a high value for the flux-to-voltage transfer function, *f*_*t*_ = |∂*V*/∂ϕ|. The latter characteristic, nonetheless, cannot be easily exploited for highly sensitive operation due to the stochastic nature of the switching, which results in an unstable working point and in a vanishing dynamic range.^36^ The transfer function, calculated through numerical differentiation of the *V*(ϕ) curves, is shown in Figure 5b for selected bias current values. The current provides an useful knob to select the flux values at which the interferometer responsivity is maximized. The maximum value of *f*_*t*_ (*f*_*t*_*M*__) is plotted versus *T* in Figure 5e. *f*_*t*_*M*__ decreases with the temperature almost linearly from the value of 400 μV/ϕ_0_ obtained at 30 mK and vanishes around 300 mK. Such a performance is on par with that of interferometers of similar typology.^36^

The impact of the gate voltage on the *V*(ϕ) was explored by repeating the acquisition of such characteristics at 30 mK as a function of both the current bias and the voltage applied to either *G*_L_ or *G*_R_. Figure 5d shows the *V*(ϕ) curves obtained for *I* = 3 μA (single lines) and *I* = 6 μA (double lines) for *V*_R_ ranging between 0 and 18 V. The first family of curves (3 μA) corresponds to a position in the parameters space where at null gate voltage the interferometer is not fully operated in the dissipative regime. The 0-voltage-drop flux interval was observed to shrink by increasing the intensity of the gate voltage until it completely disappears due to the gate-driven suppression of the critical current of the SQUID. At *V*_R_ = 18 V the device operates in a fully dissipative regime. The second family of curves (6 μA) falls entirely in the dissipative regime. We note that the result of the action of the gate is rather different from the behavior obtained by increasing the biasing current. Indeed, in the latter case both the minimum and the maximum of the modulation pattern increase by increasing the bias current. In the gate-driven regime, instead, the maximum of the modulation turns out to be locked, whereas the minimum can be controlled through the gate. These characteristics can be exploited to adapt to specific tasks the transfer function of the interferometer at the switching points through an additional knob, the gate voltage. By shrinking the width of the nondissipative region through the gate action, for example, it is possible to magnify the flux dynamic range at the switching point without reducing the overall voltage-drop swing and the resulting device sensitivity. The plot of *f*_*t*_ vs ϕ at *I* = 3 μA and *T* = 30 mK is shown in Figure 5c for several values of *V*_R_. We note that *f*_*t*_*M*__ remains almost constant in a wide gate-voltage range, as shown in Figure 5f for selected bias current values.

## Conclusions

The physics of electrostatic gating on metallic superconductors is, to date, one of the latest unanswered questions in condensed matter physics. Despite a few theoretical interpretations having been proposed, a model able to account for the totality of the phenomenology observed so far and to provide a quantitative prediction has not been developed yet. Our experiments on gated all-metallic SNS SQUIDs show that the microscopic mechanism at the origin of the critical current suppression of gated weak links is apparently phase coherent and produces a softening of the phase rigidity of the Josephson junctions. This latter observation provides a valuable reason to exclude any thermal-assimilated origin of gate-driven effects. On the other hand, we claim that among the models aiming at the description of electrostatic gating in metallic superconductors, those in which it will be possible to take into account phase coherent effects should be preferred. Here, we interpreted our data through a phenomenological model based on the sole assumption that the gate induces a phase rotation of π in the superconducting domains of the weak link subjected to the action of the electric field. Although rather simplified, our model successfully captures the main features observed in gated all-metallic SNS SQUIDs, such as the suppression of the maximum switching current, the blocking of the minimum switching current, and the deviation from the monochromatic behavior of the interferometer current–flux relation. We conclude by emphasizing the practical relevance of gated all-metallic SNS SQUIDs for magnetometry at the nanoscale. Indeed, the gate voltage provides an additional control on the transfer function of the interferometer, which can be exploited to tailor the response of the device on specific needs such as, for instance, the amplification of the flux dynamic range around the switching points for applications requiring higher sensitivity.

## Methods

### Device Nanofabrication

The SNS-SQUIDs were fabricated by a single-step electron-beam lithography (EBL) and two-angle shadow-mask metal deposition through a suspended resist mask onto an intrinsic Si(111) wafer covered with 300 nm of thermal SiO_2_. The metal-to-metal clean interfaces were realized at room temperature in an ultrahigh-vacuum (UHV) chamber (base pressure ∼ 5 × 10^–11^ Torr) of an electron-beam evaporator equipped with a tiltable sample holder. A 5 nm thick Ti adhesion film was deposited at an angle of 0°. Subsequently, 25 nm of Cu was evaporated to realize the SQUID nanowires and gates. Finally, the sample holder was tilted at 13° for the deposition of a 100 nm thick layer of Al to realize the superconducting loop.

### Cryogenic Electrical Characterization

The electrical characterization of our devices was performed by four-wire technique in a filtered cryogen-free ^3^He–^4^He dilution fridge equipped with a superconducting electromagnet, used to apply the external magnetic flux. Current–voltage (*IV*) measurement were performed by setting a low-noise current bias and measuring the voltage drop across the weak links with a room temperature preamplifier. Switching current average values were calculated over the switching points extracted from 15 repetitions of the same *IV*. The voltage–flux characterization was performed through a standard lock-in technique: the sinusoidal reference signal of the lock-in was used to current-bias the device. The in-phase output voltage signal was preamplified at room temperature. The gate voltage was applied through a room-temperature low-noise voltage source. The devices were also characterized in terms of gate-weak-link leakage current, which was found to be always lower than 1 pA.

### RSJ Fit of Experimental Data

The fitting procedure was based on eqs 1–3 together with the maximum condition . Substituting eqs 2 and 3 in eq 1, we obtain a function for the current through the loop, depending on the flux ϕ, with α, β, *I*_0_, and δ_1,2_ as parameters. The code used for the fit minimizes the distance of the function from the experimental points.
